# Active and Covert Infections of Cricket Iridovirus and *Acheta domesticus* Densovirus in Reared *Gryllodes sigillatus* Crickets

**DOI:** 10.3389/fmicb.2021.780796

**Published:** 2021-11-30

**Authors:** Kristin R. Duffield, John Hunt, Ben M. Sadd, Scott K. Sakaluk, Brenda Oppert, Karyna Rosario, Robert W. Behle, José L. Ramirez

**Affiliations:** ^1^Crop BioProtection Research Unit, Agricultural Research Service, United States Department of Agriculture, National Center for Agricultural Utilization Research, Peoria, IL, United States; ^2^School of Science, Western Sydney University, Richmond, NSW, Australia; ^3^School of Biological Sciences, Illinois State University, Normal, IL, United States; ^4^Stored Product Insect and Engineering Research Unit, Agricultural Research Service, United States Department of Agriculture, Center for Grain and Animal Health Research, Manhattan, KS, United States; ^5^Marine Genomics Laboratory, University of South Florida, St. Petersburg, FL, United States

**Keywords:** reared crickets, insects as food and feed, *Gryllodes sigillatus*, cricket viruses, entomopathogenic viruses

## Abstract

Interest in developing food, feed, and other useful products from farmed insects has gained remarkable momentum in the past decade. Crickets are an especially popular group of farmed insects due to their nutritional quality, ease of rearing, and utility. However, production of crickets as an emerging commodity has been severely impacted by entomopathogenic infections, about which we know little. Here, we identified and characterized an unknown entomopathogen causing mass mortality in a lab-reared population of *Gryllodes sigillatus* crickets, a species used as an alternative to the popular *Acheta domesticus* due to its claimed tolerance to prevalent entomopathogenic viruses. Microdissection of sick and healthy crickets coupled with metagenomics-based identification and real-time qPCR viral quantification indicated high levels of cricket iridovirus (CrIV) in a symptomatic population, and evidence of covert CrIV infections in a healthy population. Our study also identified covert infections of *Acheta domesticus* densovirus (AdDNV) in both populations of *G. sigillatus*. These results add to the foundational research needed to better understand the pathology of mass-reared insects and ultimately develop the prevention, mitigation, and intervention strategies needed for economical production of insects as a commodity.

## Introduction

Insect production is a rapidly growing industry globally. While the practice of farming insects has been around for millennia (e.g., silkworm farming and apiculture) (Defoliart, 1995), applications for mass-produced insects continue to expand beyond traditional uses (Castro-López et al., 2020; van Huis, 2020b), to include chitin production (Hahn et al., 2020), waste management and valorization (Surendra et al., 2016, 2020; Gasco et al., 2020), and use as feed for both pets, including cats and dogs (Bosch et al., 2014), and agricultural animals (Makkar et al., 2014; Henry et al., 2015; Tomberlin et al., 2015). Moreover, edible insects reared for direct human consumption (e.g., mealworms and crickets) are becoming an increasingly attractive solution to address the world’s critical need for novel and environmentally sustainable protein sources (van Huis et al., 2013; Hawkey et al., 2021). Worldwide, farmed insects have the potential to become a critically important commodity, buffering against food insecurities, providing additional revenue streams for rural and urban farmers, and offering sustainably produced resources in a growing number of applications (van Huis et al., 2013). Crickets (family: Gryllidae) are an especially popular group of insects due to their ease of rearing and nutritional profile (Wang et al., 2004; Zielińska et al., 2015; Stull et al., 2018) and are already used in many foods, including nutritional and functional additives (Hall et al., 2017; Osimani et al., 2018; Udomsil et al., 2019), and feed applications (van Huis, 2020a; Magara et al., 2021).

The demand for mass-produced insects is steadily increasing due to their utility with relatively low associated costs (Wilkie, 2018); however, there are several obstacles that hinder farmed insects from becoming an extensively utilized resource, including a dearth of rigorous empirical data (van Huis, 2017; Stull and Patz, 2020). Critical among these gaps is a lack of research on the entomopathogenic microbes that negatively impact reared insect colony health and production despite infectious disease outbreaks plaguing modern insect farms for decades (Eilenberg et al., 2018; Maciel-Vergara et al., 2021). As with traditional animal livestock, farmed insects are susceptible to parasites and pathogens, including viruses, bacteria, fungi, microsporidia, and nematodes (Kaya and Vega, 2012; Eilenberg et al., 2015). Pathogenic viruses can devastate reared insect populations (Maciel-Vergara and Ros, 2017) and especially so within cricket colonies, which are often reared in environments ideal for virus transmission (i.e., crowded, humid, and warm). For example, the *Acheta domesticus* densovirus (AdDNV), a small parvovirus, is responsible for causing severe epizootics throughout European, North American, and Asian cricket farms, resulting in massive mortality and product losses of the commonly utilized house cricket, *A. domesticus* (Styer and Hamm, 1991; Liu et al., 2011; Szelei et al., 2011; Weissman et al., 2012; Pham et al., 2013b). As a direct response to these outbreaks, many producers switched to farming alternative species, including *Gryllodes sigillatus* in North America due to reports that they were less susceptible to AdDNV (Weissman et al., 2012).

Beyond a single study (Weissman et al., 2012), we know little about viral disease and susceptibility in *G. sigillatus*, yet the list of entomopathogenic viruses infecting other farmed cricket species continues to grow to include iridoviruses, nudiviruses, and other dicistroviruses. Moreover, because there have not been wide-scale systematic surveillance efforts (de Miranda et al., 2021b), it is very likely that there are other pathogenic viruses among reared crickets still to be discovered. Here, we provide one of the first empirical reports of the identification and characterization of a viral pathogen in a diseased colony of lab-reared *G. sigillatus* crickets. We screened diseased colonies for known cricket viruses by measuring viral prevalence across sexes using both real-time PCR (qPCR) as well as Sanger sequencing and contrasted these results with a related population with no apparent signs of infection. Furthermore, we screened for novel pathogens by performing a non-targeted shotgun metagenomic analysis on guts and hemolymph from adult crickets. Metagenomic data corroborate our qPCR results and provide a complete genome of the suspected disease-causing agent, cricket iridovirus (CrIV) with significant similarities to the previously reported lizard–cricket iridovirus (Liz–CrIV) (Papp and Marschang, 2019). Our results add to the critical, but presently scarce, research on insect pathology of farmed crickets and highlight the importance of understanding viral infection and transmission dynamics in reared insect colonies.

## Materials and Methods

### Cricket Colonies

**FIGURE 1:**
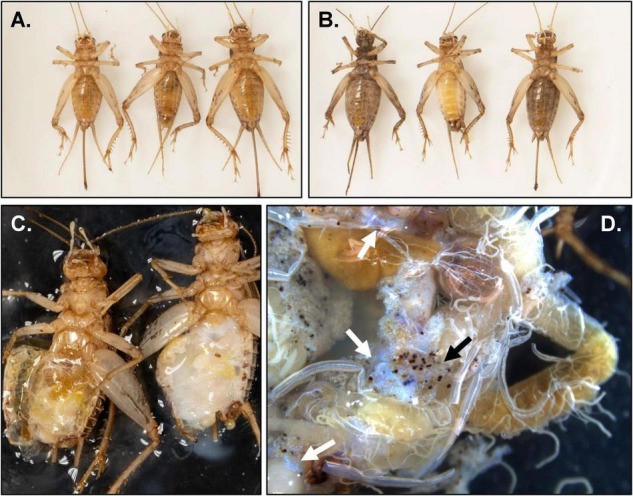
Micro- and macroscopic images of pathology discovered in adult *Gryllodes sigillatus*. **(A)** Ventral view of crickets from the Healthy population with no apparent signs of infection (from left to right: female, male, female). **(B)** Ventral view of crickets from the Diseased population with clear signs of infection (from left to right: female, male, female). **(C)** Ventral view of hemocoel of male crickets from the Healthy (left) and Diseased (right) populations. **(D)** Dissected guts from a female from the Diseased population. Note the presence of nodules (black arrow) as well as the characteristic iridescent sheen (white arrows).

Experimental *G. sigillatus* crickets came from either of two populations (“Diseased”: an apparently diseased population, or “Healthy”: an apparently disease-free population) of lab-reared colonies (20 individuals of each sex within each population; 80 crickets total). These populations were descendants from the same ancestral wild-caught crickets collected from Las Cruces, New Mexico (United States) and have been cultured in a lab setting since 2001. Populations were split and maintained in separate labs since 2007. Symptoms present in the Diseased colony were high, intermittent mortality among late-instar nymphs and adults, a strong putrid odor within rearing containers, milky white hemolymph which appeared iridescent under illuminated magnification, increased cuticle and tissue frailty, and underdeveloped or absent ovaries in some adult females (Figure 1). Rearing methods followed standard cricket rearing protocol within a research laboratory setting (Duffield et al., 2019). Briefly, about 500 crickets were housed in 55 L plastic storage bins with ventilated lids packed with egg carton to increase rearing surface area. They were provisioned with a standard diet (roughly equal parts Mazuri^®^ Rat and Mouse Diets and Purina^®^ Cat Chow Complete pellets) and water (glass vials plugged with moist cotton) *ad libitum*. All individuals were housed in an environmental chamber at 32°C on a 16 h:8 h light:dark cycle. Experimental individuals were at least 1-week-old post-eclosion when they were killed by freezing at −80°C.

### DNA, RNA Extraction, and cDNA Synthesis

To screen for DNA and RNA viruses, we extracted both DNA and RNA from whole body homogenates. Previously frozen (−80°C) crickets were placed individually in tubes with 1 mL sterile 1x PBS (pH 7.2) and two 3.2 mm diameter sterile stainless-steel beads and macerated using a TissueLyser II (Qiagen, Germany). The resulting liquid homogenate was removed (about 0.9 mL) and placed in a new sterile tube for DNA and RNA extraction.

DNA was extracted from 200 μL of cricket homogenate using the DNeasy Blood and Tissue kit (Qiagen) following the “Purification of total DNA from insects” protocol. We adapted these methods to include an addition of 200 μL of ATL buffer to the homogenate with 20 μL proteinase K and kept this mixture at 56°C overnight. RNA was extracted from 100 μL of cricket homogenate using the RNeasy Mini prep kit (Qiagen) following the “Purification of Total RNA from Animal Tissues” protocol. DNA and RNA were estimated via a NanoDrop One^C^ Microvolume UV-Vis Spectrophotometer (Thermo Fisher Scientific). All samples were stored at −20°C until further use.

### Real-Time PCR (qPCR) Detection and Quantification

For quantification purposes, we designed a primer targeting a tubulin-like reference gene (“*tubu3*,” GS-tubu-F3 5′-TGCGAGATCGTATTCCGTGG-3′ and GS-tubu-R3 5′-ACCTCGGGAGAGTCAATCCA-3′, amplicon size = 137 bp) using Primer-BLAST (NCBI) and used this as our reference gene target throughout (all primers from IDT, Inc., United States). Prior to conducting qPCR assays, we normalized DNA samples to 100 ng/μL, based on NanoDrop estimates. RNA was normalized to 1 μg, treated with DNA Wipeout, and then converted to cDNA using the QuantiTect Reverse Transcription Kit (Qiagen).

Viral screening targeted 8 known viruses identified in reared cricket populations, based on primary literature searches (Table 1): cricket iridovirus (CrIV) (Jakob et al., 2002; Papp et al., 2014), *A. domesticus* densovirus (AdDNV) (Szelei et al., 2011), *A. domesticus* mini ambidensovirus (AdMADV) (Pham et al., 2013c), *Gryllus bimaculatus* nudivirus (GbNV) (Huger, 1985; Wang et al., 2007), *A. domesticus* volvovirus (AdVVV) (Pham et al., 2013a), cricket paralysis virus (CrPV) (Wang et al., 2019), *A. domesticus* iflavirus (AdIV) (de Miranda et al., 2021b), and *A. domesticus* virus (AdV) (Valles and Chen, 2006). While most of these viruses are associated with high mortality, the pathology associated with AdMADV, AdVVV, and AdIV is currently unknown (Fernandez-Cassi et al., 2019; de Miranda et al., 2021b).

**TABLE 1:**
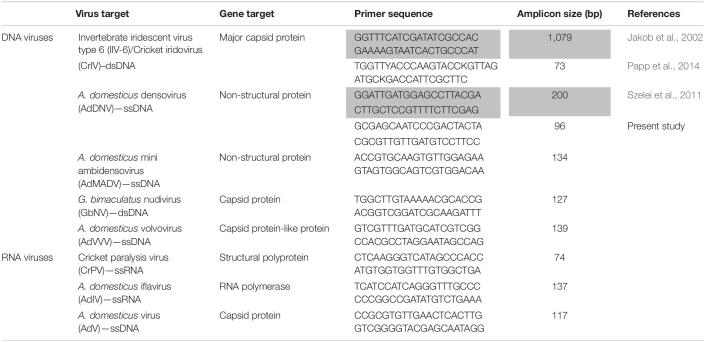
Real-time PCR (qPCR) and Sanger sequencing (in gray) primers used to target known viruses infecting rearing crickets (family: Gryllidae) in this study.

Absolute quantification was performed using known standards via double-stranded DNA (dsDNA) fragments (IDT, Inc., gBlocks Gene Fragments) designed to target the genes of interest (Table 1). The absolute abundance was expressed as the ratio of viral copies to cricket genome (*tubu3*) copies. For single stranded viruses, calculated copies were divided by 2. Real-time qPCR reactions were run on a Quant-Studio 6 Real-Time PCR instrument (Thermo Fisher Scientific, United States), and included a melt-curve stage to confirm product specificity. The identity of the viruses detected in our assays was confirmed via Basic Local Alignment Search Tool (BLAST, NCBI) following traditional PCR and Sanger sequencing (Table 1). One microliter of DNA or cDNA product was used in a 10 μL qPCR reaction using gene specific primers (Table 1) and PowerUp SYBR green Master mix kit (Qiagen). qPCR cycling conditions consisted of holding at 50°C for 2 min and 95°C for 2 min and 40 cycles of 1 s at 95°C and 30 min at 60°C. Standard curve efficiencies were 99.03% (*R*^2^ = 0.9989) for AdDNV and 91.31% (*R*^2^ = 0.9985) for CrIV. The limit of quantification for each qPCR assay was 1 copy/μL for both AdDNV and CrIV.

### Metagenomic Analysis

To screen for novel viruses, we performed non-targeted shotgun metagenomic sequencing of four adult Diseased crickets. DNA extracts from the hemolymph of each specimen and two dissected guts were used for library preparation and multiplexing using the Illumina Nextera DNA Flex kit. Briefly, libraries were prepared using the Nextera DNA flex library standard protocol according to the manufacturer’s instructions (Illumina, Nextera DNA Flex Library Prep Reference guide). Each sample was diluted to 240 ng of gDNA in 30 μL of nuclease-free water for the Tagmentation reaction, using 10 μL of Bead-Linked Transposase (BLT) and 10 μL of TB1 solution with an incubation period of 15 min at 55°C and then held at 10°C. Following Tagmentation, the DNA-BLT complex was washed three times using Tagmentation wash buffer and the tagmented DNA amplified with 5 cycles of PCR using the Enhanced PCR Mix (EPM) and Nextera DNA CD i5 and i7 index adapters (Illumina). Libraries were cleaned and 30 μL of eluted library was transferred to a new sample plate to measure their concentrations via a Quant-iT™ High-Sensitivity dsDNA Assay Kit (Thermo Fisher Scientific) on a Varioskan Lux (Thermo Fisher Scientific) microplate reader. Each sample library was diluted to 4 nM concentration, and 5 μL of the normalized library was denatured with 5 μL of 0.2 N Sodium acetate. A total of 6 Flex libraries were created (2 from gut samples and 4 from hemolymph samples). Samples were pooled and sequenced on an Illumina MiSeq system (Illumina) using a MiSeq Reagent V3 (2 × 300 bp) sequencing kit (Illumina) at the National Center for Agricultural Utilization Research (Peoria, Illinois, United States). Metagenomic data was submitted to the Sequence Read Archive (SRA) database under BioProject accession number PRJNA764167.

Metagenomic paired reads were processed using different bioinformatic applications available on the University of South Florida high performance computing cluster. Raw sequence reads were quality-filtered using Trimmomatic v 0.36.0 (Bolger et al., 2014) with default parameters. FastQC v 0.11.5 (Andrews, 2010) was used to verify the quality of trimmed sequences and assemblies were performed using metaSPAdes v 3.11.1 with default parameters (Nurk et al., 2017). Assembled contigs were filtered by size on the Galaxy web-based platform (Afgan et al., 2018) to retain contigs larger than 100 bp. Contig sequences were compared against the GenBank non-redundant database using BLASTx as implemented in DIAMOND (Buchfink et al., 2015) to identify viral sequences.

BLAST searches revealed large contigs (∼195 kb) with significant similarities to an iridovirus (Liz-CrIV; accession number MN081869) in each of the six libraries. These contigs were further explored using Geneious Prime v 2021.1.1 given that their size approximated near-complete iridovirus genomes. Since iridovirus genomes can be circularly permutated (Jakob et al., 2001), putative unit length genomes were obtained by identifying terminally redundant sequences for annotation purposes. To evaluate genome coverage, quality-trimmed forward reads from each library were mapped to the identified unit length iridovirus genomes using default parameters within the “Map to Reference” Geneious tool. Genome co-linearity was verified using Mauve with the progressiveMauve algorithm (Darling et al., 2010) as implemented in Geneious Prime. A representative viral genome, named cricket iridovirus isolate Liz-CrIV_USDA_2019 (CrIV_USDA), was submitted to GenBank under accession number OK181107. Genome-wide pairwise identities among genomes were calculated by aligning sequences using EMBOSS Stretcher (Madeira et al., 2019). Alignments were then used to calculate pairwise identities using the formula employed by the Species Demarcation Tool, which has been used to classify viral sequences (Muhire et al., 2014). Specifically, the percent identity values were calculated as [1-(M/A)]*100 where M is the number of mismatching nucleotides and A the total number of aligned positions with no gaps.

### Statistical Analysis

To compare viral copy abundance across populations and between sexes, we performed general linear models for the ratio of viral copies normalized to cricket *tubu3* copies including population (Diseased or Healthy), sex, and virus (CrIV or AdDNV) and their interactions. Individuals with viral copies lower than the limit of quantification were removed from analysis. All abundance data were log transformed to fit normality assumptions and reported results derive from the best models as determined by corrected Akaike’s information criterion (AICc using the stepAIC function in R; Sugiura, 1978; Hurvich and Tsai, 1989) or before the removal of terms from the final model. All statistical analyses were carried out in R (version 4.0.5, R Core Team, 2021) and graphs were made using GraphPad Prism 9 (version 9.0.0). Viral prevalence and abundance data can be accessed in Supplementary Material Data Sheet 1.

## Results

### Viral Prevalence and Abundance via qPCR

Across 80 crickets sampled, we detected CrIV and AdDNV in all but one individual for each virus (both females from the Healthy population, 98.75% positivity rate for each target) (Table 2). Based on qPCR, we did not find any evidence that either population was infected with additional DNA (AdMADV, GbNV, AdVVV) or RNA viruses (CrPV, AdIV, AdV) (Table 2).

**TABLE 2:** Prevalence of targeted viruses across two populations (“Healthy” and “Diseased”) of male and female adult reared *Gryllodes sigillatus* using qPCR.

Virus target		Diseased population		Healthy population	
		Females	Males	Females	Males
DNA viruses	Cricket iridovirus (CrIV)	20/20 (100%)	20/20 (100%)	19/20 (95%)	20/20 (100%)
	*A. domesticus* densovirus (AdDNV)	20/20 (100%)	20/20 (100%)	19/20 (95%)	20/20 (100%)
	*A. domesticus* mini ambidensovirus (AdMADV)	0/80 (0%)			
	*G. bimaculatus* nudivirus (GbNV)	0/80 (0%)			
	*A. domesticus* volvovirus (AdVVV)	0/80 (0%)			
RNA viruses	Cricket paralysis virus (CrPV)	0/80 (0%)			
	*A. domesticus* iflavirus (AdIV)	0/80 (0%)			
	*A. domesticus* virus (AdV)	0/80 (0%)			

**FIGURE 2:**
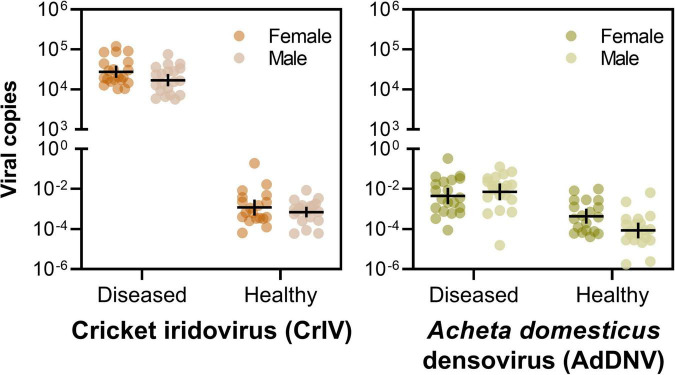
Viral loads of cricket iridovirus (CrIV) (left) and *Acheta domesticus* densovirus (AdDNV) (right) detected across two populations (“Diseased” and “Healthy”) of male and female adult reared *Gryllodes sigillatus* (geometric mean ± 95% confidence intervals). The graphs depict the ratio of viral copies normalized to cricket *tubu3* copies, shown on a log scale. *Post hoc* comparisons are indicated on Table 4.

Notably, crickets from the Diseased population of both sexes had a much greater abundance of CrIV copies compared to their Healthy counterparts and significantly more copies of CrIV than AdDNV in both populations (Tables 3, 4 and Figure 2). Males and females had similar viral loads of CrIV across both populations. For AdDNV, males and females from the Diseased population had similar viral loads while males had significant lower viral loads compared to females within the Healthy population (Tables 3, 4 and Figure 2). Both male and female crickets from the Diseased population also had a greater abundance of AdDNV compared to the Healthy population (Tables 3, 4 and Figure 2). Estimated amounts of viral copies per cricket are listed in Supplementary Table 1.

**TABLE 3:** Model terms and statistics from generalized linear models for log-transformed absolute abundance (number of viral copies) of cricket iridovirus (CrIV) and *Acheta domesticus* densovirus (AdDNV) detected across two populations (“Healthy” and “Diseased”) of male and female adult reared *Gryllodes sigillatus*.

Model term	*F*	df	*P*
**Population**	**1503.20**	**1**	**<0.0001**
**Sex**	**4.28**	**1**	**0.0404**
**Virus**	**1013.97**	**1**	**<0.0001**
**Population × Sex**	**4.12**	**1**	**0.0441**
**Population × Virus**	**674.91**	**1**	**<0.0001**
Sex × Virus	0.02	1	0.8750
*Population × Sex × Virus*	*3.88*	*1*	*0.0506*

*Bold terms denote statistical significance (α = 0.05).*

**TABLE 4:** *Post hoc* comparisons of interactions from generalized linear models for log-transformed normalized number of viral copies of cricket iridovirus (CrIV) and *Acheta domesticus* densovirus (AdDNV) detected across two populations (“Healthy” and “Diseased”) of male and female adult reared *Gryllodes sigillatus*.

Contrast			Estimate ± SE	Z ratio	*P*
Population (Diseased vs. Healthy)	CrIV	Females	**16.97 ± 0.528**	**32.14**	**<0.0001**
		Males	**17.00 ± 0.521**	**32.62**	**<0.0001**
	AdDNV	Females	** 2.31 ± 0.528**	**4.37**	**<0.0001**
		Males	** 4.41 ± 0.521**	**8.45**	**<0.0001**
Sex (Males vs. Females)	CrIV	Diseased	0.486 ± 0.521	0.93	0.3516
		Healthy	0.517 ± 0.528	0.98	0.3279
	AdDNV	Diseased	−0.466 ± 0.521	−0.89	0.3715
		Healthy	**1.633 ± 0.528**	**3.09**	**0.0020**
Virus (CrIV vs. AdDNV)	Female	Diseased	−**15.64 ± 0.521**	−**30.01**	**<0.0001**
		Healthy	−0.98 ± 0.535	−1.83	0.0669
	Male	Diseased	−**14.69 ± 0.521**	−**28.19**	**<0.0001**
		Healthy	−**2.10 ± 0.521**	−**4.02**	**0.0001**

*Bold terms denote statistical significance (α = 0.05) following Tukey adjustment for multiple comparisons.*

### Genome Sequence of CrIV via Shotgun Metagenomic Analysis

**FIGURE 3:**
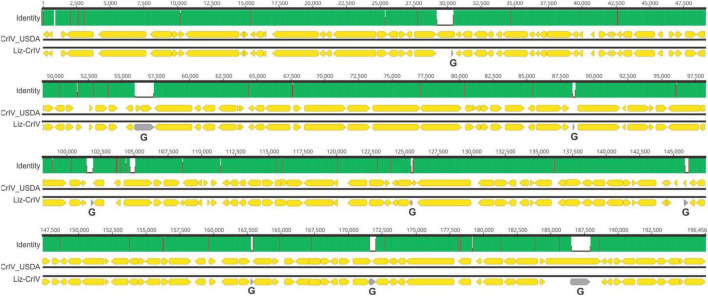
Schematic showing a genome-wide pairwise comparison between CrIV_USDA and lizard–cricket iridescent virus (Liz-CrIV). Each block, including “Identity,” “CrIV_USDA,” and “Liz-Cr-IV,” illustrates the shared identity and organization for each genome, respectively. The numbers at the top each block indicate alignment positions. The “Identity” panel highlights identical sites (green), mismatches (red), and gaps (white) across the alignment. Non-overlapping open reading frames encoding putative proteins larger than 80 amino acids in each genome are highlighted with yellow arrows. Areas of the Liz-CrIV genome annotated as gaps (G) in GenBank are highlighted with gray arrows.

Metagenomic analysis confirmed the presence of CrIV across all samples. We identified near-complete CrIV genomes with high coverage in each sample (mean coverage ranged from 53x to 1050x), suggesting high viral concentrations. These genomes shared >99.9% identity (genome size between individuals ranged from 194,811 to 195,316 bp). Genome-wide pairwise identities revealed that CrIV isolated from the Diseased population, referred herein as “CrIV_USDA”, was most closely related to Liz-CrIV (accession no. MN081869) (97%) (Figure 3) followed by Invertebrate iridescent virus 6 (IIV6) (accession no. NC_003038) (71.5%) (Supplementary Figure 1). We were not able to detect AdDNV in our metagenomic analysis which could be due to the fact that we had low concentrations in our samples and AdDNV is a ssDNA virus with a relatively small genome (5,425 bp) (Liu et al., 2011; de Miranda et al., 2021a).

## Discussion

Entomopathogenic viruses are known to cause significant losses to the reared insect industry (Maciel-Vergara and Ros, 2017); however, little is known about their diversity, biology, and host association. Using a range of molecular approaches in the present study, we were able to identify an cricket iridovirus (CrIV) as the likely causal agent of a disease affecting a colony of reared *G. sigillatus*, a species of growing importance for food, feed, and industrial purposes (Weissman et al., 2012). Metagenomic analyses revealed that our CrIV isolate, CrIV_USDA, was most closely related to lizard-cricket iridovirus (Liz-CrIV) (Papp et al., 2014; Papp and Marschang, 2019). Gross pathology included milky hemolymph, decreased fecundity, sluggish behavior, and melanotic lesions, all indicative of an active microbial infection. Our molecular identification and quantification assays indicated that Diseased crickets were supporting large numbers of CrIV. Diseased crickets were estimated to have about 1.5 trillion more copies of CrIV than Healthy crickets, based on the volume of DNA template extracted and used in our qPCR assays (Supplementary Table 1). Although our studies did not include a complete Koch’s postulate to confirm the causal agent infecting this colony of *G. sigillatus*, our molecular characterization based on shotgun metagenomics of hemolymph and gut samples and qPCR-based absolute quantification strongly suggests that CrIV is the main viral entomopathogen driving the pathology in the Diseased population. Future studies will focus on dissecting the intricacies of host-pathogen interactions, routes of transmission, and identifying the biological factors that might be associated with an active and covert infection. The latter consideration will be essential as we do not yet fully understand why CrIV presents as an active infection in one population but not the other.

Invertebrate iridescent viruses (family *Iridoviridae*) have non-occluded icosahedral particles, approximately 130 nm diameter, that contain a double-stranded DNA genome of about 140–210 kpb (Williams et al., 2000; İnce et al., 2018). They can infect a broad range of invertebrates, including terrestrial isopods, and have been isolated from several insect taxa (Kleespies et al., 1999; Jakob et al., 2002). Furthermore, certain invertebrate iridoviruses are known to cause disease in reptiles (Just et al., 2001; Weinmann et al., 2007; Marschang, 2011; Papp et al., 2014) and amphibians (Stöhr et al., 2016) via ingestion of infected insects, posing a particular health concern for the pet trade industry. In crickets, CrIV is known to cause disease in several species, including reared *Gryllus texensis* (Adamo et al., 2014), *G. bimaculatus* (Just and Essbauer, 2001), *G. campestris*, *A. domesticus* (Kleespies et al., 1999), and now *G. sigillatus*.

Our metagenomic analyses confirm the draft genome of Liz-CrIV from a previous study (Papp and Marschang, 2019) as a distinct virus from Invertebrate Iridescent virus 6 (IIV-6), although formal analyses are needed to determine if Liz-CrIV represents a strain of IIV-6 or a new species of invertebrate iridovirus (Papp and Marschang, 2019). Analyses from the current study also provide the most complete genome of Liz-CrIV. Importantly, our PCR primers were not able to discern between IIV-6 and Liz-CrIV and so future screening efforts should target areas of the genome that distinguish the two (see Supplementary Figure 1). Liz-CrIV was first discovered in Europe from commercially produced crickets in the mid to late 1990’s (Kleespies et al., 1999; Just and Essbauer, 2001). The populations used in the present study, descents of field-caught crickets in New Mexico, have been reared in a research lab setting since 2001, and have had no history of contact with commercially produced crickets. While we currently do not know the origins or the spread of this virus, our study confirms that Liz-CrIV is active across multiple continents.

In addition to active CrIV infections, our study found evidence for covert, or asymptomatic, infections of both CrIV and *Acheta domesticus* densovirus (AdDNV), the latter of which has been documented in *G. sigillatus* previously (Weissman et al., 2012). Covert infections of invertebrate iridovirus are reportedly more prevalent than active lethal infections in some insect populations (Williams, 1993, 2008; Tonka and Weiser, 2000). Although our study design did not allow us to examine in detail the effects that covert infections might have on cricket health, covert invertebrate iridovirus infections may have significant fitness consequences for hosts (e.g., increased development times and reduced fecundity) (Marina et al., 1999, 2003), which could have important ramifications for product yield in production facilities. Our study design prevents us from distinguishing between a persistent infection (with low levels of virus replication) or a latent infection and additional experiments (e.g., measuring transcription) could elucidate the nature of these covert infections (Williams et al., 2017).

Interestingly, we found a significant effect of the interaction between sex and population on the abundance of viral copies such that Healthy males had a lower abundance of AdDNV copies compared to Diseased males and females from either population. Although females are the larger sex (Sakaluk et al., 2019), our analysis normalized abundance values such that body size would not account for these differences. Thus, our results could be indicative of a higher tolerance of AdDNV by females, but controlled exposures would be needed to confirm this. We did not see the same sex-effect for CrIV. Few studies, if any, quantify viral loads across sex in reared and farmed insects, which makes generalizing this finding difficult. However, previous studies have demonstrated higher immune activity in female *G. sigillatus* compared with males (Gershman et al., 2010), which could contribute to differences in viral loads.

A key aspect of this system yet to be determined is the route of virus transmission, although *per os* is suspected to be the main route for CrIV (Williams et al., 2005). Adamo et al. (2014) found that CrIV can be transmitted horizontally via topical exposure in *G. texensis* and found no evidence of virus within the testes. Similar results were found by Just and Essbauer (2001) who found no signs of infection in the ovaries or testes in *G. bimaculatus*. However, both studies assessed transmission among populations experiencing active infections and so it is plausible that CrIV may be transmitted vertically when populations are exhibiting covert infections. Indeed, several classes of pathogenic viruses, including iridoviruses, are known to adopt mixed-mode transmission (both vertical and horizontal transmission) based on the relative fitness gains that are obtained via each transmission strategy (Ebert, 2013). Because horizontal transmission is riskier when hosts are rare but vertical transmission is constrained by host fitness, this mixed-mode transmission balances the likelihood of transmission with the constraints of host fitness. Thus, vertical transmission is favored when host densities are low while horizontal transmission is favored when host density is high. Further studies exploring the potential for mixed-mode transmission of CrIV across populations with active and covert infections will be critical and may explain the discrepancy between populations.

In conclusion, foundational research on the pathology of mass-produced insects, including crickets, will be essential to maintain the health and yield necessary for this growing industry. Here, we report the viral loads across sexes of active and covert infections of two pathogenic viruses (CrIV and AdDNV) in two populations of reared *G. sigillatus*. Importantly, these findings will inform future work addressing diagnostic, mitigation, and therapeutic interventions in reared insect colonies that could ultimately improve product yield and support this burgeoning sustainable industry.

## Data Availability Statement

Full genome of the CrIV isolate (Cricket iridovirus isolate Liz-CrIV_USDA_2019) can be found at GenBank (submission number: OK181107). Metagenomic sequences for hemolymph and guts are available at the NCBI Sequence Read Archive database (study number: PRJNA764167, SRA accession numbers: SAMN21469590, SAMN21469591, SAMN21469592, SAMN21469593, SAMN21469594, and SAMN21469595). The datasets presented in this study can be found in online repositories. The names of the repository/repositories and accession number(s) can be found in the article/Supplementary Material.

## Author Contributions

KD and JR contributed to conception, design of the study, and collected the data. KR and BO analyzed metagenomic data. KD performed statistical analyses and wrote the first draft of the manuscript. KR and JR wrote sections of the manuscript. BS, SS, JH, and RB provided necessary samples, materials, and additional expertise. All authors contributed to manuscript revision, read, and approved the submitted version.

## Conflict of Interest

The authors declare that the research was conducted in the absence of any commercial or financial relationships that could be construed as a potential conflict of interest.

## Publisher’s Note

All claims expressed in this article are solely those of the authors and do not necessarily represent those of their affiliated organizations, or those of the publisher, the editors and the reviewers. Any product that may be evaluated in this article, or claim that may be made by its manufacturer, is not guaranteed or endorsed by the publisher.
