# Supplementary material for: Active and Covert Infections of Cricket Iridovirus and Acheta domesticus Densovirus in Reared Gryllodes sigillatus Crickets
Source: Front Microbiol. 2021 Nov 30;12:780796. doi: 10.3389/fmicb.2021.780796 (PMC8670987; doi:10.3389/fmicb.2021.780796)
Supplement: Supplementary file 1 [file Table_1.DOCX]

Table S1: Average estimated number of copies of virus per individual based on a) DNA volume and b) normalized amounts of DNA used in RT-qPCR reaction found in *Gryllodes sigillatus* crickets from two populations.

| ***a) Based on volume of DNA template used in RT-qPCR reaction*** | | | |
| --- | --- | --- | --- |
| **Population** | **Sex** | **Average estimated copies of CrIV/cricket** | **Average estimated copies of AdDNV/cricket** |
| *Diseased* | Female | 1.48E+12 | 2.31E+05 |
|  | Male | 1.53E+12 | 1.40E+05 |
| *Healthy* | Female | 2.43E+05 | 5.09E+04 |
|  | Male | 2.03E+06 | 1.99E+04 |
|  | | | |
| ***b) Based on normalized amount of DNA used in RT-qPCR reaction*** | | | |
| **Population** | **Sex** | **Average estimated copies of CrIV/cricket** | **Average estimated copies of AdDNV/cricket** |
| Diseased | Female | 4.45E+11 | 2.20E+05 |
|  | Male | 4.75E+11 | 4.41E+05 |
| Healthy | Female | 9.68E+04 | 2.18E+04 |
|  | Male | 7.45E+05 | 5.51E+03 |


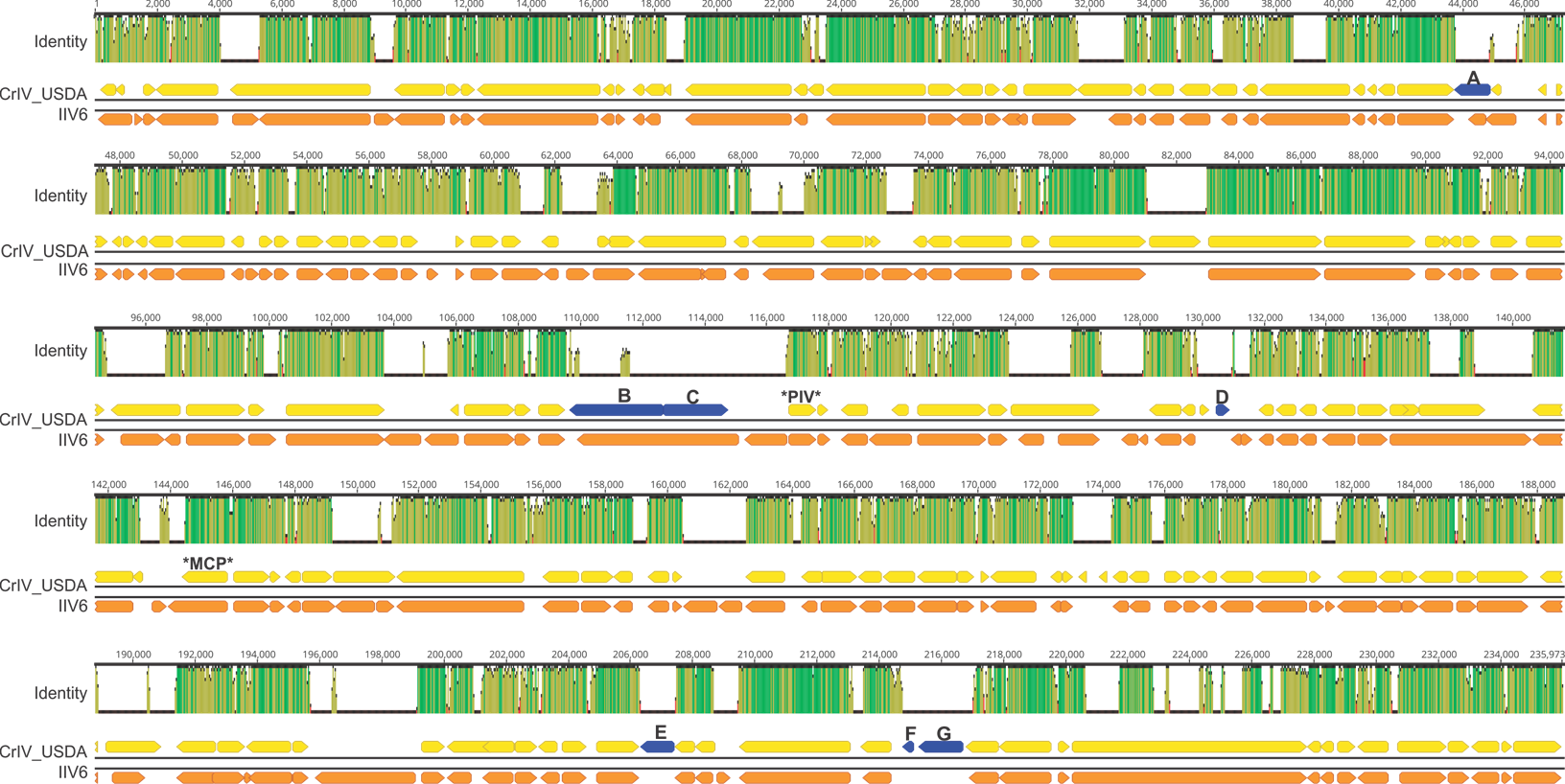


Figure S1. Schematic showing a genome-wide pairwise comparison between CrIV_USDA and invertebrate iridescent virus 6 (IIV6). Each block including ‘Identity’, ‘CrIV_USDA’, and ‘IIV6’ illustrates the shared identity and organization for each genome, respectively. The numbers indicate alignment positions. The ‘Identity’ panel highlights mean pairwise identities in a given region. Identical regions are highlighted in green, mean identities over 30% in green-yellow and those below 30% in red. Non-overlapping ORFs encoding putative proteins larger than 80 amino acids are highlighted with yellow and orange arrows for CrIV_USDA and IIV6, respectively. ORFs unique to the Liz-CrIV genome are highlighted in blue, including those encoding putative proteins most similar to IIV31_BRO-like (A), IIV31_128L (B), IIV31_015L (C), E3 ubiquitin ligase (D), IIV31_084R (E), IIV31_074L (F), and dUTPase (G) proteins. Sites for primers used to detect IIV6, including those targeting genes encoding the major capsid (MCP) and thymidylate synthase (PIV) proteins, are indicated.
